# Vitamin D in adolescent idiopathic scoliosis: a meta-analysis

**DOI:** 10.1186/s12891-023-06793-0

**Published:** 2023-08-29

**Authors:** Dong Suk Kim, Jung Sub Lee

**Affiliations:** Department of Orthopaedic Surgery, Biomedical Research Institute, School of Medicine, Pusan National University Hospital, Pusan National University, 179 Gudeok-Ro, Seo-Gu, Busan, 49241 Republic of Korea

**Keywords:** Vitamin D, Adolescent, Idiopathic scoliosis, Meta-analysis

## Abstract

**Objective:**

The aim of this study was to compare serum vitamin D levels in girls with adolescent idiopathic scoliosis (AIS) and controls using meta-analysis methods. We searched Medline (via PubMed), Cochrane, Scopus, and Embase databases for studies evaluating outcomes in AIS, including patient age, body mass index, bone mineral density (BMD), and serum levels of parathyroid hormone (PTH), calcium, and phosphate, published between January 2000 and June 2020. We searched for studies that were limited to humans only. The inclusion criteria were a scoliosis study that measured vitamin D levels. We excluded duplicate publications such as review articles, case reports, and letters without original data. Two authors extracted data independently and resolved any discrepancies by consensus.

**Results:**

Eight comparative studies were identified. Demographic characteristics, bone density, serum levels of vitamin D, parathyroid hormone, and phosphate levels were not significantly different between AIS group and controls, except for serum calcium levels. The serum calcium levels were lower in AIS group than in the controls.

**Conclusions:**

This review includes eight comparative studies reporting serum vitamin D and/or parathyroid hormone levels in AIS. Due to heterogeneity, a limited number of meta-analyses have shown a weak correlation between serum vitamin D levels and the incidence of AIS. Larger, multicenter studies are therefore needed to validate the results.

**Supplementary Information:**

The online version contains supplementary material available at 10.1186/s12891-023-06793-0.

## Introduction

Adolescent idiopathic scoliosis (AIS) is a three-dimensional spinal deformity that primarily affects adolescent girls [1, 2]. Despite numerous studies over the decades, the cause of AIS remains unknown [1, 2]. The causes of AIS are generally known to be multifactorial [1, 2]. The onset and progression of scoliosis affect spinal growth in many ways [3]. Low body mass index (BMI), low body weight, tall stature, long arm length, delayed menarche, and low total body bone mass [4, 5] are factors that have been associated with AIS in previous studies. AIS is also associated with systemic disorders. For instance, scoliosis may be associated with deformity, low BMI, abnormal skeletal growth, and low bone mineral status.

Previous studies suggest that vitamin D may play an essential role in the cerebral process of postural balance. Serum vitamin D levels are positively correlated with hip bone mineral density (BMD) and negatively correlated with Cobb angle. In addition, sufficient level of vitamin D is an important component for musculoskeletal development, maintenance, and function [6–9]. Thus, vitamin D insufficiency or deficiency affects the etiopathogenesis of AIS. While some case-controlled studies have documented impaired vitamin D levels in patients with AIS, others have not found a significant association [9–16]. Therefore, this study performed a meta-analysis and focused on the factors showing the association between vitamin D levels and the risk of AIS.

## Materials and methods

### Data search and study selection

In this study, we searched databases published in English in Medline (via PubMed), Cochrane, Scopus, and Embase from January 2000 to June 2020. In addition, to use a broad category of papers, we used a search algorithm that included the following terms: “scoliosis”, “adolescent idiopathic scoliosis”, “AIS”, “vitamin D”, “25-hydroxyvitamin D”, “25 (OH) vitamin D”, “dihydroxycholecalciferol”, “parathyroid hormone”, “PTH”, “calcium” and “phosphorus”. We followed the standard PRISMA guidelines for conducting meta-analyses and wrote the manuscript according to the PRISMA checklist [17] (see Supplement 1).

### Study selection

First, we looked for studies that were limited to humans. The inclusion criteria were a scoliosis study that measured vitamin D levels. Duplicate publications such as review articles, case reports, and letters without original data were excluded. Each of the two researchers reviewed the titles and abstracts of the retrieved articles that met the above criteria. Articles with clear ineligible factors were rejected. The same two researchers then examined the full text of these articles to assess their eligibility for inclusion. All discrepancies were resolved by consensus.

### Data extraction

The following information was extracted from each study: [1] article information including authors, year of publication, country of origin, study design, and patient characteristics; [2] primary outcome including serum 25(OH)D (25-OHD) level; and [3] secondary outcome presented as serum PTH, calcium and phosphate levels, BMI and BMD.

### Assessment of study quality

We used the Newcastle-Ottawa scale to assess the methodological quality of the case-control studies [18]. The scale consists of nine items covering the following three dimensions: [1] patient selection (four items); [2] comparability of the two study arms (two items); and [3] outcome assessment (three items). The total score ranges from 0 to 9, with higher scores indicating better quality. In this study, a score of 6 or more indicates a study of high quality.

### Data synthesis and analysis

We used Review Manager (RevMan version 5.2, Copenhagen: The Nordic Cochrane Centre, The Cochrane Collaboration, 2012) to analyze the data from each study. Odds ratios and their 95% confidence intervals (CIs) were calculated for dichotomous variables. For continuous variables, we calculated the mean difference and 95% CIs, while the results of all studies were performed on the same scale. Heterogeneity between studies was assessed using χ[2] tests and I [2] statistics [19]. Publication bias was assessed using the effective sample size funnel plot [20]. Two-sided p ≤ 0.05 was considered statistically significant.

## Results

### Literature search and selection of studies

**Fig. 1:**
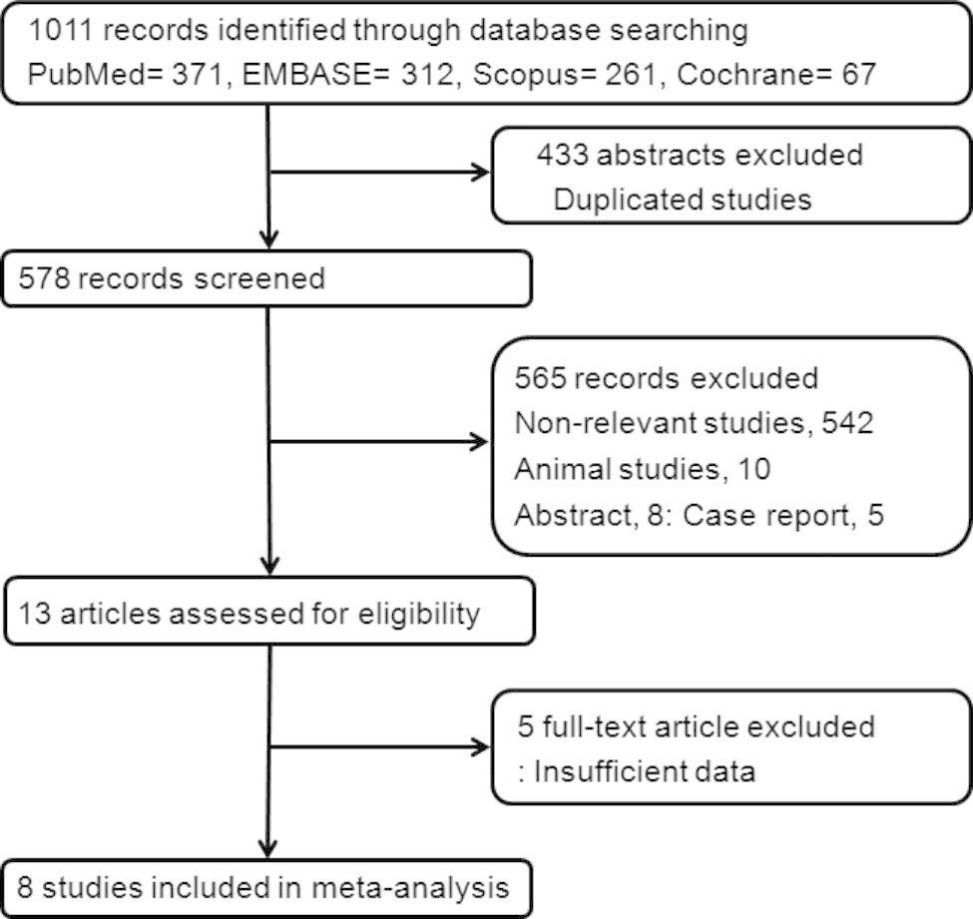
Flowchart of the study selection process

1,011 records were obtained through extensive computerized searches and intensive cross-validations of reference lists, and 433 publications among them were excluded due to the similarities of the title and abstract. In addition, 542 non-relevant studies, 10 animal studies, 8 abstracts, and 5 case reports were excluded. The remaining 13 full-text articles met the qualification. Finally, eight studies were included in the systematic review and meta-analysis, presented in the reference Sects. [9–16]. The detailed procedure for selecting studies for meta-analysis is shown in Fig. 1. The patient demographics data of the studies are shown in Table 1.

**Table 1:** Characteristics of the included studies

Authors	Subject number Scoliosis/Control	Age (years) Scoliosis/Control	Sex Scoliosis(F:M)/Control(F:M)	Cobb’s angle	Design	NOS score
Ahuja	30 / 12	13.4 / 12.9	15:15 / 6:6	49.4	Case-control study Level of evidence: III	7
Akseer	30 / 19	24.8 / 23.5	30:0 / 19:0	36.5	Cross-sectional study Level of evidence: III	8
Balioglu	229 / 389	14.7 / 13.9	177:52 / 167:222	-	Retrospective study Level of evidence: III	7
Batista	55 / 60	20 / 13.6	- / -	-	Prospective study Level of evidence: III	8
Catan	32 / 32	14.75 / 14.75	32:0 / 32:0	31	Prospective study Level of evidence: II	8
Gozdzialska	100 / 100	13.61 / 12.77	100:0 / 100:0	-	Cross-sectional study Level of evidence: III	8
Lam	212 / 183	12.9 / 12.9	- / -	-	Unclear	6
Suh	198 / 120	12.5 / 12.7	198:0 / 120:0	-	Prospective study Level of evidence: II	8

NOS, Newcastle-Ottawa Scale

### Patient characteristics

**Fig. 2:**
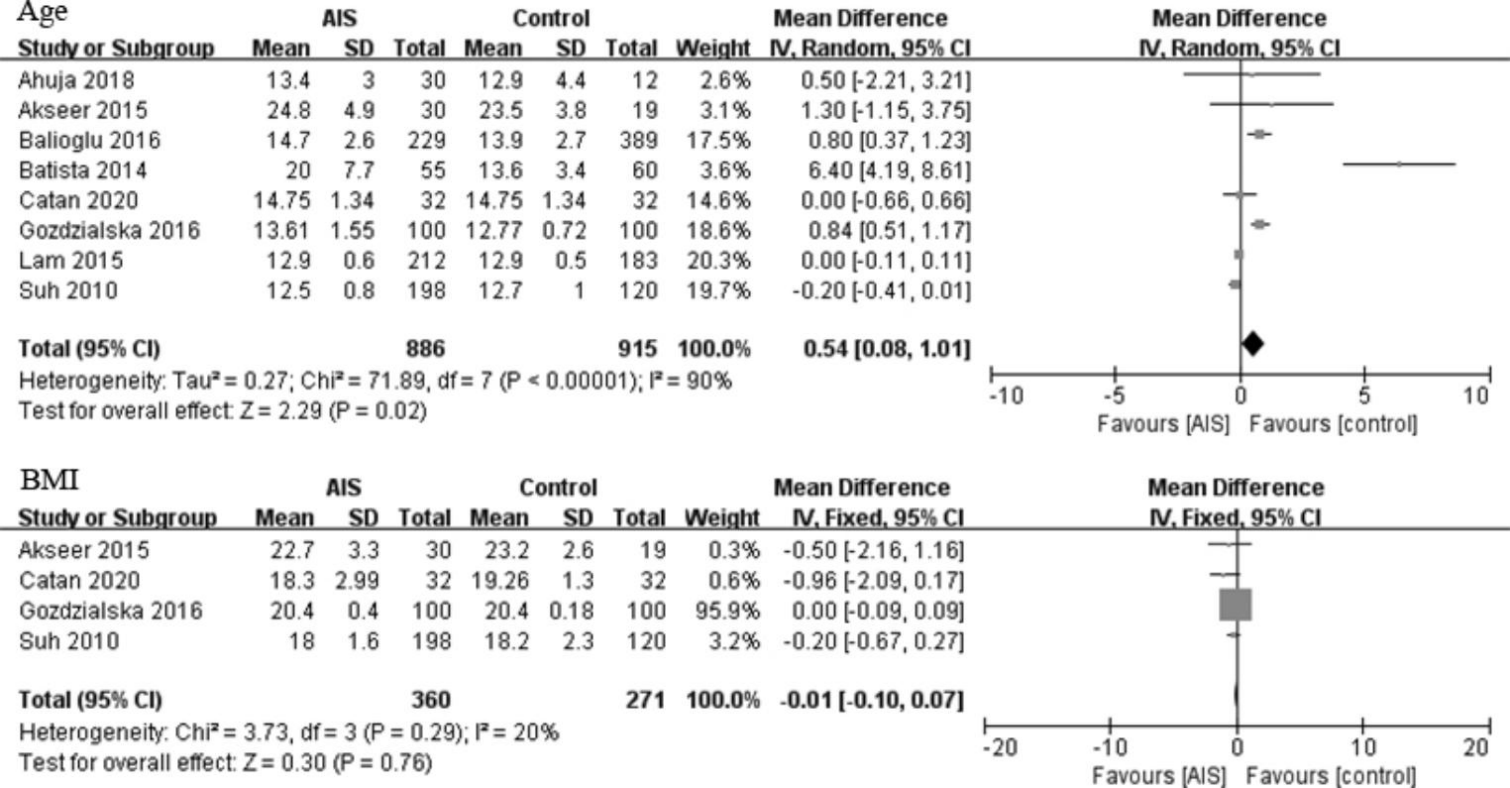
Forest plots of the patients’ characteristics between the AIS and control groups

The mean age of the AIS and control groups was 15.8 and 14.6 years, respectively. The AIS group was older than the control group (p = 0.02; weighted mean difference [WMD] = 0.54 [0.08, 1.01] years; Fig. 2). The mean BMI of the AIS and control group was 19.9 and 20.3 kg/m^2^, respectively (p = 0.79, WMD = 0.01 [− 0.10, 0.07] kg/m^2^; Fig. 2).

### Serum 25-OHD and PTH levels

**Fig. 3:**
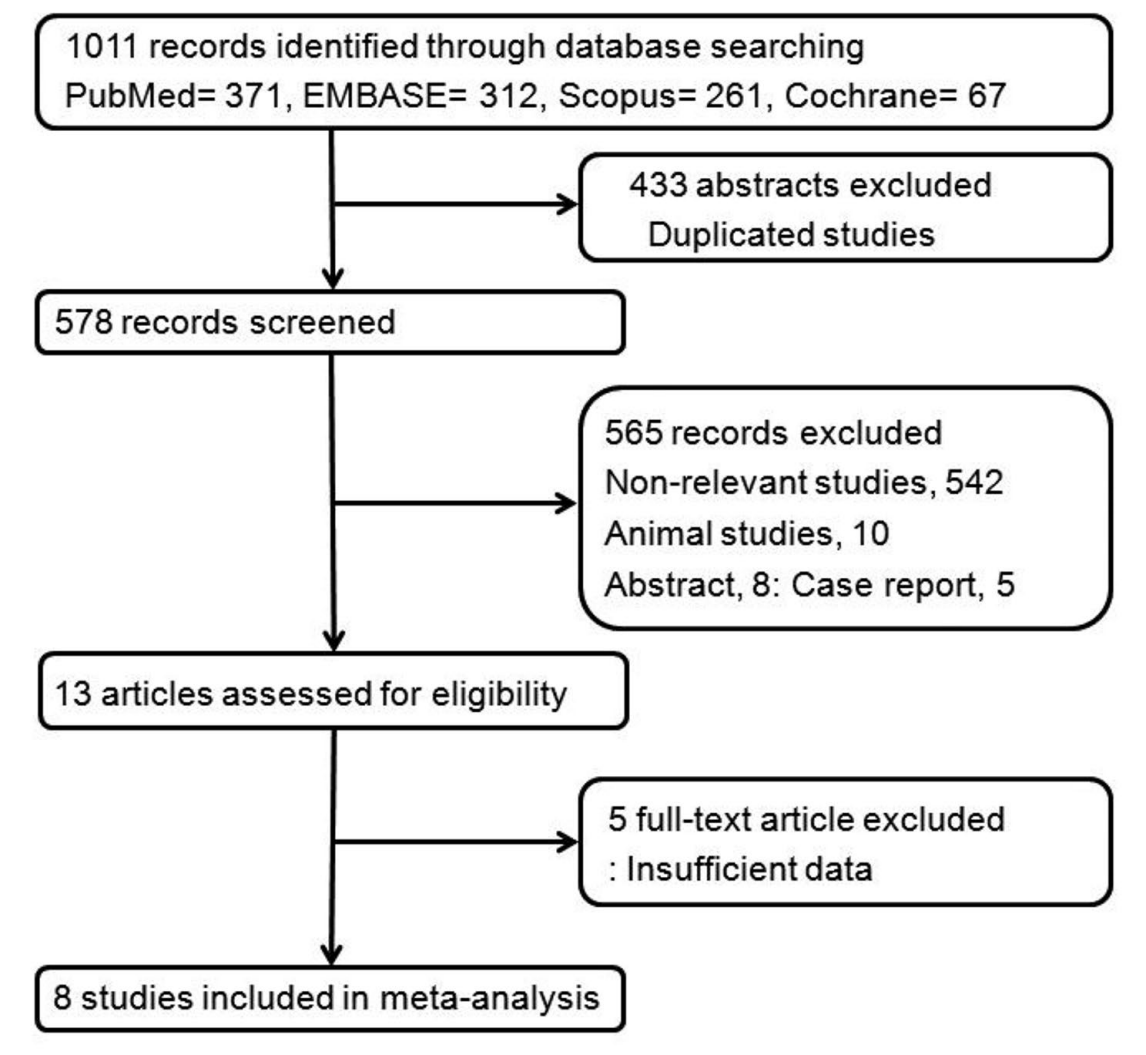
Forest plots of the 25-OHD and PTH levels between the AIS and control groups

The mean serum 25-OHD levels of the AIS and control group were 21.0 and 26.6 ng/mL, respectively, with no significant difference between the two groups (p = 0.08, WMD=-5.58 [− 11.88, 0.72] ng/mL; Fig. 3). The mean PTH levels in the AIS and control group were 43.8 and 38.8 pg/mL, respectively (p = 0.96, WMD = 0.09 [-3.45, 3.63] pg/mL; Fig. 3). There was no significant difference found in the PTH level between the two groups (Fig. 3).

### BMD and serum calcium and phosphate levels

**Fig. 4:**
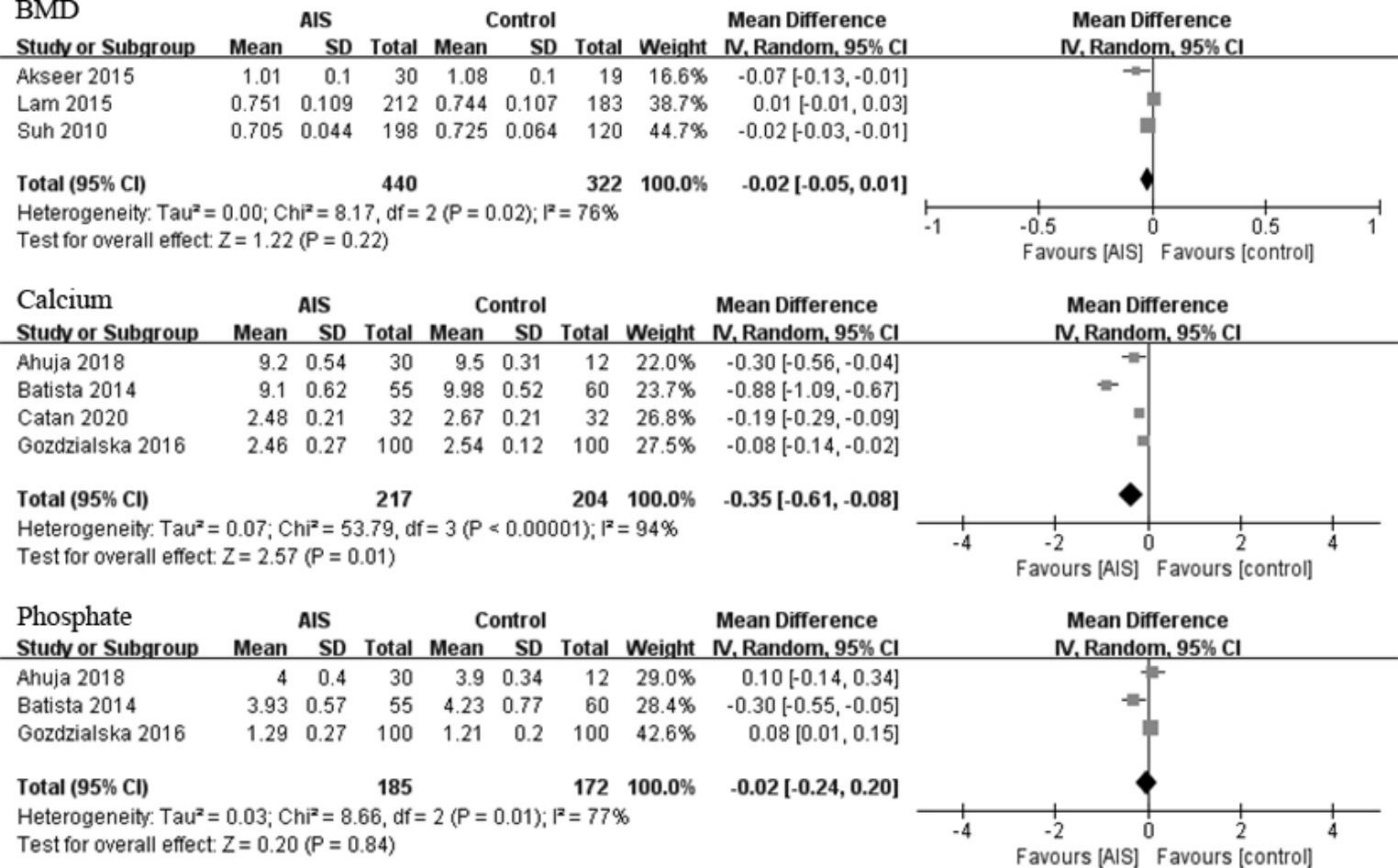
Forest plots of the BMD, calcium and phosphate levels between the AIS and control groups

Femoral neck BMDs of the AIS and control group were 0.822 and 0.850 g/cm^2^, respectively, with no significant difference between the two groups (p = 0.22, WMD = − 0.02 [− 0.05, 0.01] g/cm^2^; Fig. 4). In addition, serum phosphate levels of the AIS and control group were 3.1 and 3.1 mg/dL, respectively, with no significant difference between the two groups (p = 0.84, WMD = − 0.02 [− 0.24, 0.20] mg/dL; Fig. 4). However, the serum calcium levels of the AIS and control group were 5.8 and 6.2 mg/dL, respectively (p = 0.01, WMD = − 0.35 [− 0.61, -0.08] mg/dL; Fig. 4), indicating a significantly higher level in the control group.

## Discussion

Girls with AIS have low BMI, abnormal skeletal growth, and relative anterior spinal overgrowth with uncoupled neuro-osseous growth. These factors are intricately connected to bone metabolism. Previous studies have discussed the impact of vitamin D on spinal growth, which can potentially contribute to the development of spinal scoliotic deformities [21–23].

Vitamin D plays a crucial role in maintaining calcium phosphate balance within the body. Consequently, a deficiency in vitamin D can potentially lead to not only bone abnormalities but also other diseases [24, 25]. In addition, insufficient levels of vitamin D may impact the development of AIS by influencing fibrosis regulation, postural control, and bone metabolism. Several previous studies have suggested lower vitamin D levels in individuals with AIS compared to healthy individuals. However, the association between AIS and alterations in parathyroid hormone (PTH) and vitamin D levels remains a topic of ongoing debate and lacks a consensus among studies [9, 11, 12] In this meta-analysis, we investigated the relationship between vitamin D levels and susceptibility to AIS.

This meta-analysis included 8 comparative studies, which examined the relationship between vitamin D levels and Adolescent Idiopathic Scoliosis (AIS). The findings of the meta-analysis indicated that the AIS group had slightly lower vitamin D levels compared to the control group. However, the difference between the two groups was not statistically significant (p = 0.08, weighted mean difference [WMD]=-5.58 [-11.88, 0.72] ng/mL). These results suggest a weak association between vitamin D deficiency and the development of AIS. Nevertheless, further studies are necessary to understand the underlying mechanism of AIS. The regulation of calcium phosphate homeostasis involves the hormone parathyroid hormone (PTH) and vitamin D. Our study did not find any statistical differences in PTH levels, bone mineral density (BMD), or serum phosphate levels between the AIS and control groups. However, the calcium levels were lower in the AIS group compared to the control group. Although the exact mechanism for this decrease in serum calcium levels remains unknown, it aligns with previous studies that reported low bone mass and osteopenia throughout the axial and peripheral skeleton in AIS [4, 5, 26−28].

There are certain limitations in our study. Firstly, the sample sizes of the eight trials included in this meta-analysis were relatively small, potentially leading to increased heterogeneity and bias. Despite efforts to address this issue by incorporating studies with varying participants, inconsistent inclusion criteria, and different treatments, heterogeneity may still persist. Furthermore, we did not conduct subgroup analyses to account for factors that could contribute to heterogeneity. Therefore, interpretation of the pooled data should be approached with caution [29] Large, well-designed studies are needed to provide high-quality evidence on the association between serum vitamin D levels and AIS.

The present meta-analysis shows that there was no statistical difference in vitamin D and PTH levels between the AIS and control groups, while serum calcium levels were lower in AIS patients. However, it is important to note that both the control group and AIS group have serum calcium levels that fall within the range of hypocalcemia. This study, being a meta-analysis aimed at determining statistically significant values, suggests the need for further research to explore the clinical implications of these findings. Therefore, further large multicentre studies are needed to confirm our results.

### Electronic supplementary material

Below is the link to the electronic supplementary material.

**Supplement 1**: The Preferred Reporting Items for Systematic Rewiews and Meta-Analyses (PRISMA) 2020 checklist

## Data Availability

All the data generated or analyzed in this study has been incorporated into this paper.
